# Mechanical characterization data of polyacrylamide hydrogel formulations and 3D printed PLA for application in human head phantoms

**DOI:** 10.1016/j.dib.2023.109114

**Published:** 2023-04-05

**Authors:** Anthony J.A. Baker, Eric J. Galindo, James D. Angelos, Dustin K. Salazar, Sorcha M. Sterritt, Adam M. Willis, Michaelann S. Tartis

**Affiliations:** a New Mexico Institute of Mining and Technology, Department of Chemical Engineering, 801 Leroy Place, Socorro, NM 87801, USA; b Michigan State University, Department of Mechanical Engineering, East Lansing MI, 48824, USA; c 59th Medical Wing, Office of the Chief Scientist, Lackland AFM, TX, 78236, USA

**Keywords:** Brain phantom, Hydrogel, Brain tissue, Polylactic acid (PLA), Skull, Polyacrylamide (PAA), 3D Printing, Mechanical testing, Rheology

## Abstract

To study human traumatic brain injury (TBI) mechanics, a realistic surrogate must be developed for testing in impact experiments. In this data brief, materials used to simulate brain tissue and skull are characterized for application in a full-scale human head phantom. Polyacrylamide hydrogels are implemented as tissue scaffolds and tissue mimics because they are bioinert and tunable. These properties make them ideal for use as brain tissue in studies that simulate head impacts. The objective is to modify hydrogel formulations to have minimal swelling and optical clarity while maintaining properties that mimic brain tissue, such as density, viscoelastic properties, and rheological properties. Secondly, polylactic acid (PLA) polymers are 3D printed to create biomimetic skulls to enclose the hydrogel brain tissue mimic or brain phantom. PLA samples are printed and tested to determine their mechanical strength with the intention of roughly matching human skull properties. Hydrogel data was obtained with an oscillatory rheometer, while PLA samples were tested using a mechanical tester with a 3-point bend setup. The present data brief highlights several hydrogel formulations and compares them to identify the benefits of each formula and reports mechanical values of 3D printed PLA samples with 100% grid infill patterns applied in a skull model.

**Specifications Table**

**Table:** 

Subject	Biomaterials; Material Characterization
Specific subject area	Biomimetic material fabrication; Hydrogel characterization; 3D Printed Materials; 3D Printed Polymer characterization
Type of data	Graph Figure
How the data were acquired	Rheological data is obtained on a stress-controlled rheometer, Stress Rheometer 5 (SR5), with a parallel plate geometry using the Thermal Analysis (TA) Orchestrator software v7.2.0.4. The upper fixture plate size is 25 mm. Samples were placed on the lower fixture and the upper fixture was lowered onto the sample, then dynamic frequency sweeps were performed. Optical values were assessed quantitatively with UV-vis spectroscopy to determine clarity. Three-point bend testing was performed on a Mark 10 ESM1500S model following ASTM standards with different crosshead speeds of 3.41 mm/min and 10 mm/min with a 6.3 mm roller diameter. To fabricate the 3D printed samples for mechanical testing a Creality CR-10S Pro V2 printer was used with filament from Hatchbox 3D Filaments.
Data format	Raw Analyzed
Description of data collection	PAA hydrogels were analyzed after reaching equilibrium in an isotonic solution, as this was the state of interest. The excess solution was removed with a paper towel before testing. The Peltier plate temperature for rheological testing was set at 21°C with a tolerance of ± 0.5°C. Samples were excluded if they showed signs of hyperelastic behavior, slippage, or if temperature drifted beyond ± 1°C. In 3-point bend testing, PLA samples were 154 mm in length, 12.7 mm in width, and 8 mm in thickness, as the upper roller approached the sample at 10 mm/minute or 3.4 mm/minute. UV-vis spectroscopy readings were obtained after swelling hydrogels. Hydrogels were placed in a 1 cm cuvette and tested from 400 – 700 nm in 2 nm increments.
Data source location	All testing was performed at the following location: Institute: New Mexico Institute of Mining and Technology City: Socorro, NM 87801 Country: United States of America Longitude and latitude: 34.06655842341045, -106.90564025666204 Hydrogel polymerization, rheology testing, and 3D printing occurred in the Chemical Engineering department and 3-point bend testing was performed in the Physics department.
Data accessibility	Repository name: Mendeley Data Data identification number: DOI: 10.17632/c3rxvdgbrs.1 Direct URL to data: https://data.mendeley.com/datasets/c3rxvdgbrs Repository name: Mendeley Data Data identification number: DOI: 10.17632/gfpmzx49mx.1 Direct URL to data: https://data.mendeley.com/datasets/gfpmzx49mx
Related research article	Knutsen, A.K., Vidhate, S., McIlvain, G., Luster, J., Galindo, E.J., John- son, C.L., Pham, D.L., Butman, J.A., Mejia-Alvarez, R., Tartis, M., Willis, A.M.: Characterization of material properties and deformation in the angus phantom during mild head impacts using MRI. Journal of the Mechanical Behavior of Biomedical Materials 138, 105586 (2023). https://doi.org/10.1016/j.jmbbm.2022.105586

## Value of the Data

• The data presented characterizes equilibrated PAA hydrogel formulations tuned to match *ex vivo* brain tissue and *in vivo* human brain magnetic resonance elastography data. These formulations may be utilized in other tissue modeling applications.
• The data characterizes the flexural strength of a 3D printed PLA skull model with 100 % grid infill.
• Variations of hydrogel formulations exhibited in the data may be utilized as tissue scaffolds for cell types that are responsive to substrate stiffness for differentiation.
• This brief provides a foundation to utilize these hydrogels as a tissue scaffold and to further characterize these PAA hydrogels at the microscale.
• The brief addresses issues in the literature regarding the high variation of rheological data in hydrogels and the limited data on hydrogels with high polymer content.

## 1 Objective

This data brief supports the publication titled *‘Characterization of Material Properties and Deformation in the ANGUS Phantom During Mild Head Impacts’* by providing the foundational mechanical characterization data necessary to fabricate a head phantom with specified properties to analyze simulated mild head impacts [1]. This data brief characterizes two cranial components, brain tissue and skull. PAA hydrogels are used as a biomaterial because they are bioinert and tunable [2]. In the literature, hydrogel properties and formulations are highly variable making it difficult to reproduce. Additionally, limited data is available on hydrogels with high polymer content. Variations in monomer and crosslinker content, temperature, and linear acrylamide chains provide different properties that can meet desired specifications, such as swelling, optical clarity and rheological properties. Monomer to crosslinker formulations of 60-1 provide optical clarity but swell significantly, while 15-1 formulations have minimal swelling but are turbid. An ideal formulation will minimally swell and remain optically clear for high-speed imaging. These formulations, for white and gray matter, achieve a storage modulus of 2000 or 1400 Pa, a loss modulus of 400 or 300 Pa, respectively, and a tan δ of 0.2 [1,3]. Hydrogels with increased linear acrylamide chains were formulated in an effort to obtain an increased viscous component thereby increasing the tan δ, the ratio of viscous to elastic forces [4]. An important property of the skull is its ability to deform before it fails, the flexural modulus is analyzed in 3-point bend tests on PLA samples with 100% grid infill to mimic skull behavior.

## 2 Data Description

Two categories are reported, PAA characterization to represent brain tissue and 3D printed PLA parts to represent the skull in the human head surrogate. The rheology, optical, and swelling data are all contained in the ‘*Hydrogel Rheology, Optical Values, and Swelling’* repository [5]. Figures 1-3 display rheological data, the file names in the repository describe which formulation was used by specifying the acrylamide weight percent and crosslinker ratio. In this brief, weight percent is written as wt. % and is defined as the percent weight per volume. The file name also specifies samples that were heated or used linear acrylamide chains [5]. UV spectroscopy and hydrogel swelling data are also included. The UV spectroscopy excel file has each sample in an individual sheet and includes the wavelength and absorbance values, the file name, and the time and date on which the test was conducted [5]. Lastly, the swelling data is included in an excel file that has the time in hours which is highlighted in green. The average and standard deviation values of the three dimensions and the volume are calculated for each time point in centimeters [5]. Three Python scripts are also included in the repository, two rheology scripts, and one swelling script. The two rheology scripts and MATLAB script were hard queried for certain data files, all of which are included in the repository. Both rheology scripts average the desired data, and all scripts used the matplotlib library for visualization.

The ‘*3-Point Bend Testing for 100% Grid Infill PLA Samples’* data repository includes three files, two excel files which contain six sheets. The first five sheets are the individual samples that are collected including their load, travel, time, and calculated values for the load versus displacement and flexural modulus [6]. The sixth sheet contains the average and standard deviation for all samples. The associated MATLAB script evaluates one excel file at a time and using known values of depth, support span, and width calculates strain and stress based on calculations obtained from ASTM D790 [6,7].

Fig. 1 presents the average a) storage, b) loss, and c) tan δ values obtained in a dynamic frequency sweep comparing 60-1 and 15-1 crosslinker to monomer formulations with 5-12 wt. % monomer content. Hydrogel raw data sets, available in the data repository associated with this article, show that most formulations had n = 10 with the exceptions of 5 wt. % 15-1 (n = 8) and 10 wt. % 15-1 (n = 9) [5]. The excluded samples in those two formulations were due to hyperelastic behavior caused by a stress setting beyond the yield stress of the sample. Fig. 1a shows the average storage modulus of 60-1 versus 15-1 formulations at different monomer (wt. %) content where 15-1 hydrogels with similar monomer content provide a higher storage modulus. A similar comparison is apparent in the loss modulus (Fig. 1b). The tan δ for 60-1 and 15-1 formulations is in Fig. 1c. Both formulations obtain a range of rheological properties desired for tissue scaffold and brain mimic applications. Based on Magnetic Resonance Elastography of healthy volunteers and indentation measurements of the bovine brain it was determined that an ideal hydrogel formulation to mimic storage and loss modulus of brain tissue would be 2000 Pa and 400 Pa for white matter and 1400 Pa and 300 Pa for gray matter, respectively, that would result in a tan δ of 0.2 [1,3]. Fig. 1 was created with the Python script, ‘*Rheology – Hydrogel Monomer and Crosslinker Comparison’* which was used to analyze six files *(Rheology_10_wt_15_1, Rheology_10_wt_60_1, Rheology_12_wt_15_1, Rheology_5_wt_15_1, Rheology_7_wt_15_1, Rheology_7_wt_60_1).* All these files are included in the data repository titled ‘*Hydrogel Rheology, Optical Values, and Swelling’* [5].

**Fig. 1a:**
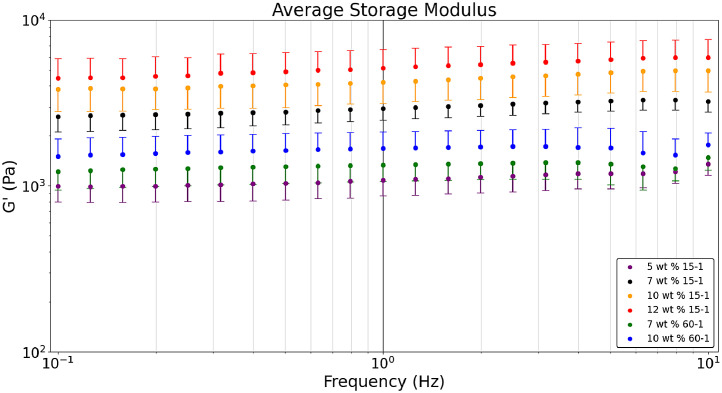
Dynamic frequency sweeps comparing the average storage modulus of hydrogel formulations with crosslinker ratios of 15-1 versus 60-1 at 5-12 wt. % monomer content.

**Fig. 1b:**
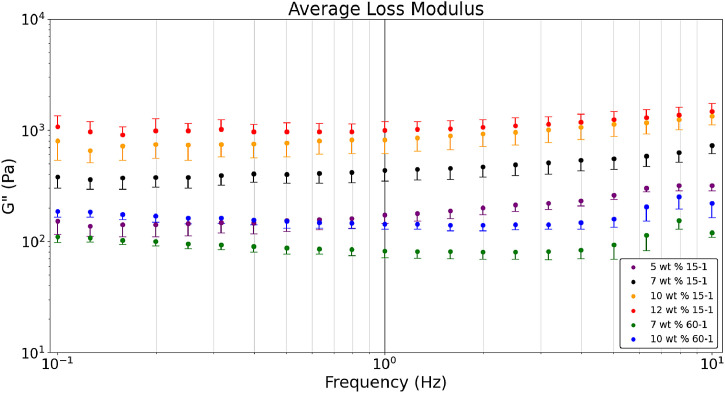
Dynamic frequency sweeps comparing the average loss modulus of hydrogel formulations with crosslinker ratios of 15-1 versus 60-1 at 5-12 wt. % monomer content.

**Fig. 1c:**
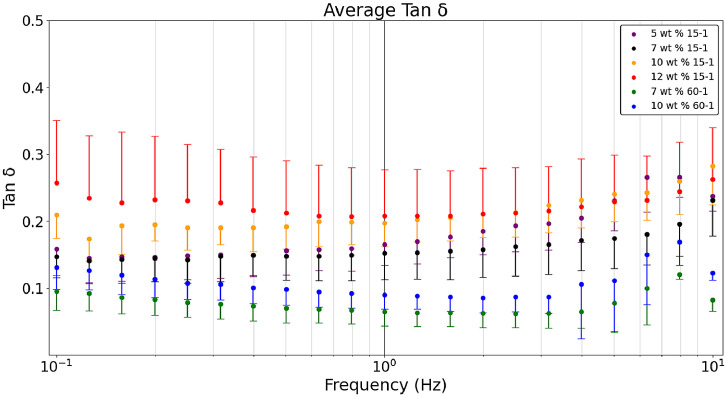
Dynamic frequency sweeps comparing the average tan δ of hydrogel formulations with crosslinker ratios of 15-1 versus 60-1 at 5-12 wt. % monomer content.

Fig. 2 displays the average a) storage, b) loss, and c) tan δ values obtained in a dynamic frequency sweep (n = 10) for linear acrylamide formulations. Linear acrylamide is created with the intent of producing longer acrylamide chains not bound to a crosslinker that produces a dampening effect and thereby an increase in the loss modulus. A higher tan δ value was expected with the addition of linear acrylamide chains because of its higher viscosity compared to DI water [4]. These chains are created by pre-polymerizing the monomer into longer chains before crosslinking (see Linear Acrylamide Hydrogel Formulations in Methods). Raw data sets corresponding to Fig. 2 are available in the data repository associated with this article [5]. Fig. 2 compares 60-1 and 15-1 monomer to crosslinker ratios with 10 wt. % monomer content and 15 wt. % linear acrylamide chains hydrogel formulations. Figs. 2a and 2b demonstrate that higher crosslinker formulations (15-1) provide higher storage and loss values compared to lower crosslinker formulations (60-1) as expected. Fig. 2c displays that the tan δ of linear acrylamide hydrogels is lower than that of 60-1 and 15-1 formulations without linear acrylamide chains. Fig. 2 was created with the Python script, *‘Rheology_LinearAnalysis’* which analyzes two files *(Rheology_10_wt_15_1_15linear, Rheology_10_wt_60_1_15linear)*. Both files are included in the data repository titled *‘Hydrogel Rheology, Optical Values, and Swelling’* [5].

**Fig. 2a:**
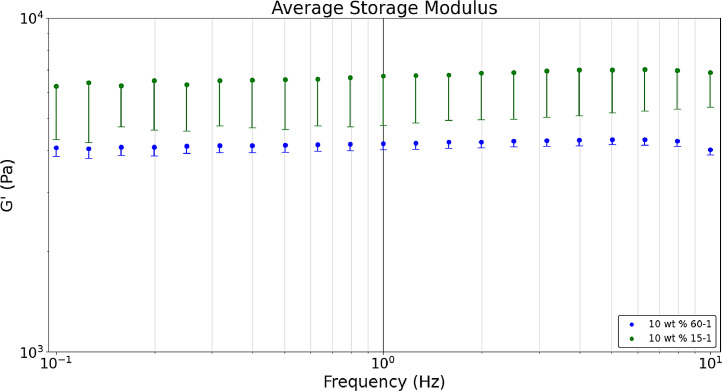
Dynamic frequency sweeps comparing the average storage modulus of two linear acrylamide hydrogel formulations, both with 15% linear acrylamide, at crosslinker ratios of 60-1 and 15-1 and 10 wt. % monomer content.

**Fig. 2b:**
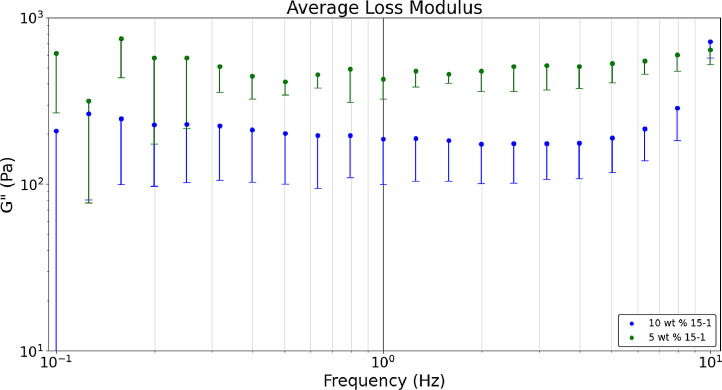
Dynamic frequency sweeps comparing the average loss modulus of two linear acrylamide hydrogel formulations, both with 15% linear acrylamide, at crosslinker ratios of 60-1 and 15-1 and 10 wt. % monomer content.

**Fig. 2c:**
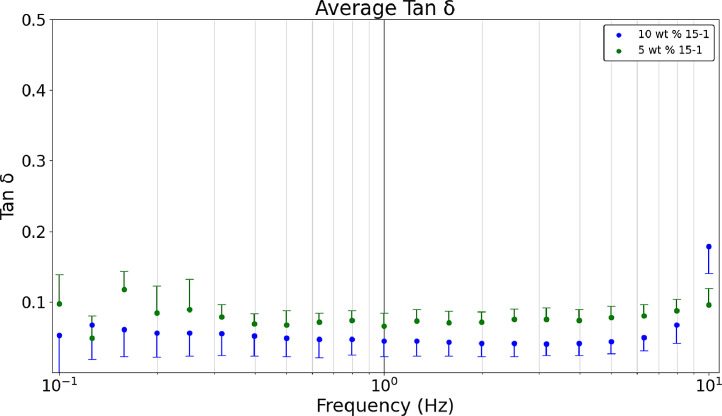
Dynamic frequency sweeps comparing the average tan δ ratio of two linear acrylamide hydrogel formulations, both with 15% linear acrylamide, at crosslinker ratios of 60-1 and 15-1 and 10 wt. % monomer content.

Fig. 3 shows the average a) storage, b) loss, and c) tan δ values obtained in a dynamic frequency sweep (n = 9). Raw data sets corresponding to Fig. 3 are available in the data repository associated with this article [5]. Fig. 3 formulations are comparing heated hydrogel formulations of 10 wt. % and 5 wt. % acrylamide and 15-1 crosslinker ratio. Polymerizing hydrogels at 60°C dissociates the initiator (ammonium persulfate) into free radicals meaning bonds are broken which results in gels that are more optically clear and have lower storage and loss modulus values than non-heated formulations [8]. The tan δ value of 0.2 was near the desired value. Fig. 3 was developed with the Python script, *‘Rheology – Hydrogel Monomer and Crosslinker Comparison’* which analyzes two files, *‘Rheology_Heated_Hydrogels_10wt_15_1’* and *‘Rheology_Heated_Hydrogels_5wt_15_1’* both of which are included in the data repository titled *‘Hydrogel Rheology, Optical Values, and Swelling’* [5].

**Fig. 3a:**
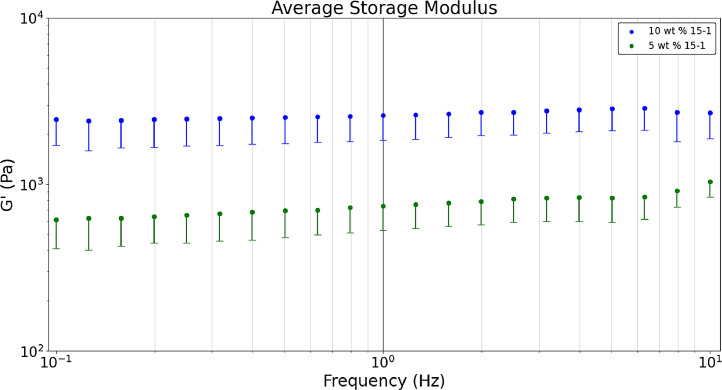
Dynamic frequency sweeps comparing the 5 wt. % 15-1 and 10 wt. % 15-1 heated hydrogels’ average storage modulus.

**Fig. 3b:**
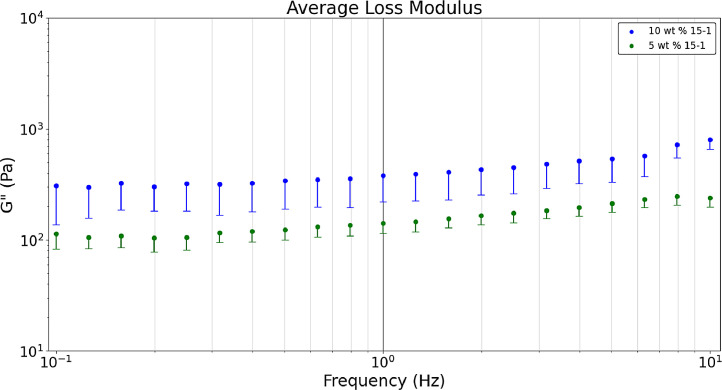
Dynamic frequency sweeps comparing the 5 wt. % 15-1 and 10 wt. % 15-1 heated hydrogels’ average loss modulus.

**Fig. 3c:**
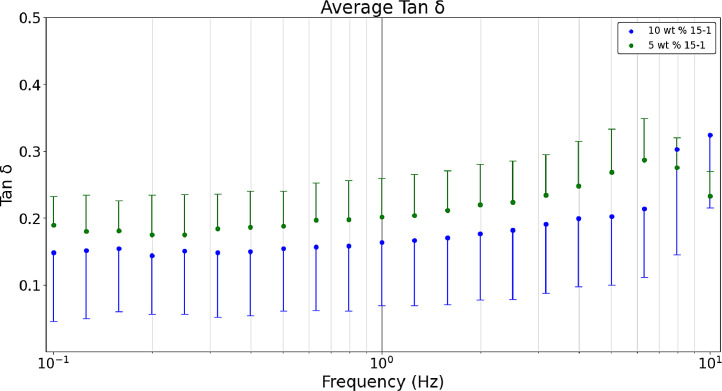
Dynamic frequency sweeps comparing the 5 wt. % 15-1 and 10 wt. % 15-1 heated hydrogels’ average tan δ values.

**Fig. 4:**
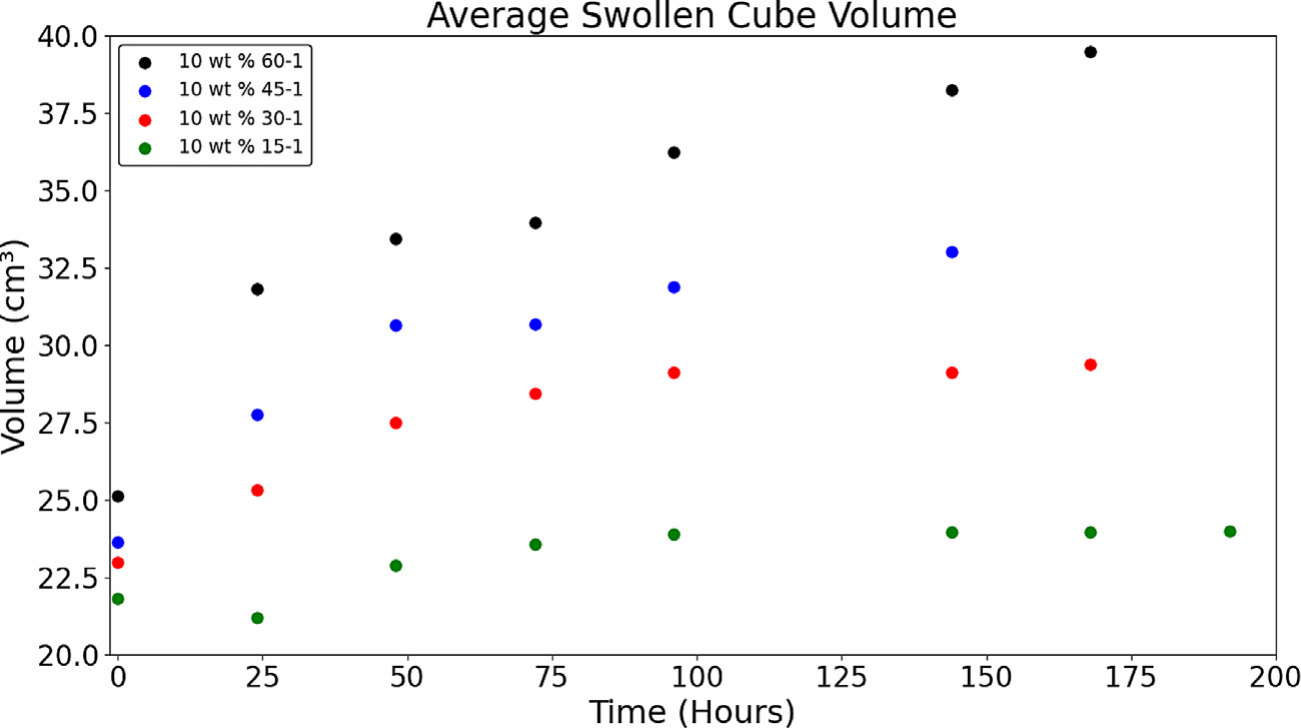
Average volume overtime for different crosslinker ratios at the same monomer weight percentage. On day 6 averages were as follows: 60-1 – 38.24 ± 0.068 cm^3^; 45-1 – 33.01 ± 0.042 cm^3^; 30-1 – 29.13 ± 0.109 cm^3^; 15-1 – 23.98 ± 0.045 cm^3^.

Fig. 4 displays the difference in swelling between different crosslinker ratios, 15-1 provides the least amount of swelling while 60-1 has the most. There are minor deviations in measurement because of user error, average volume was used to monitor swelling and changes in dimensions were measured using digital calipers. 60-1, 45-1, and 15-1 formulations had n = 6 and the 30-1 formulation had n = 4. Fig. 4 was visualized with a Python script titled *‘Swelling_10wt’* which analyzed the file *‘Swelling_Data’* included in the data repository titled *‘Hydrogel Rheology, Optical Values, and Swelling’* [5].

**Fig. 5:**
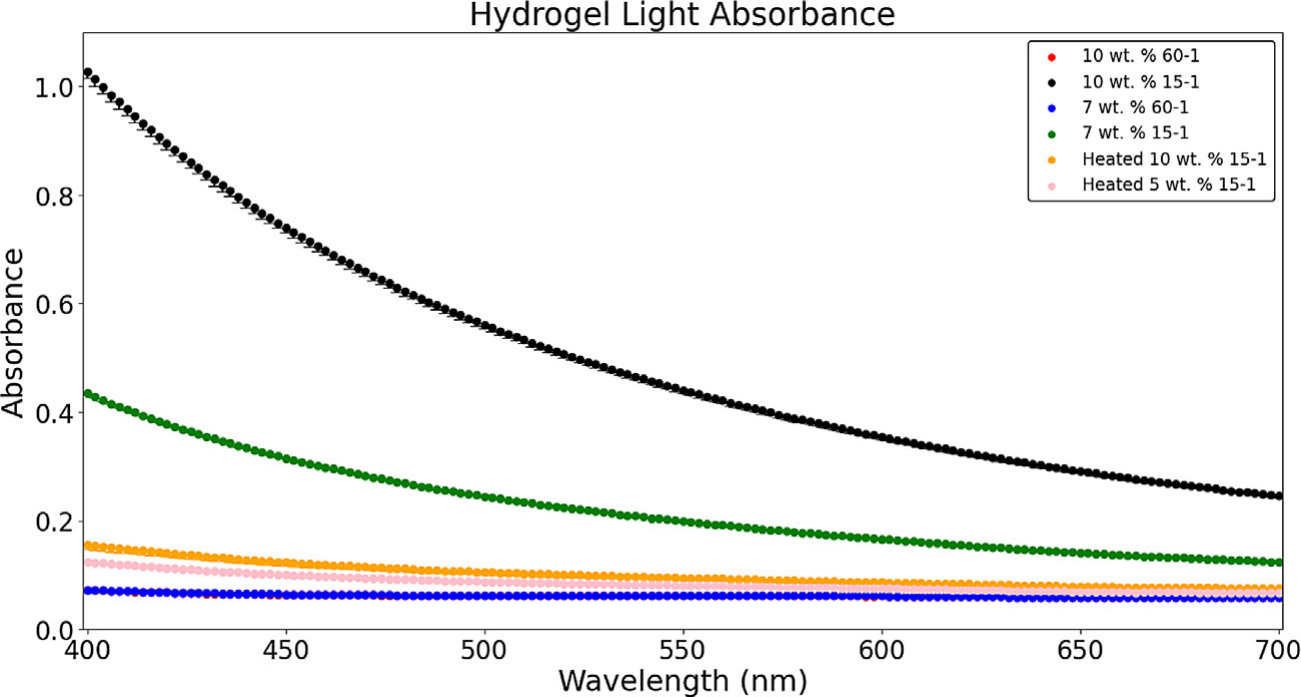
Comparison of the average light absorbance in hydrogels (n = 3) at crosslinker ratios of 60-1 and 15-1, including hydrogels polymerized at 60°C.

Fig. 5 compares different hydrogel formulations’ light absorbance with a path length of 1 cm from the file *‘UV_Vis_Reading´* included in the data repository *‘Hydrogel Rheology, Optical Values, and Swelling’* [6]. Higher crosslinker formulations (15-1) had more light absorbance than lower crosslinker formulations (60-1) because of the higher density of bonds in 15-1 formulations. The 10 wt. % 60-1 is hidden behind the 7 wt. % 60-1, this stems from the large absorbance axis limits to include the 15-1 data and also because of the similar clarity in 60-1 formulations at these monomer values. However, when 15-1 formulations were polymerized at 60°C instead of room temperature light absorbance was lower than 15-1 formulations and more comparable to 60-1 formulations because of the dissociation of the initiator into free radicals [8].

Fig. 6 shows the average flexural stress for PLA samples with a 100% grid infill (n = 5) used in the skull of brain phantoms, with the ‘X’ signifying the fracture point [1]. Literature values for the flexural modulus of human skull is reported at 11.73 GPa [9]. Samples were tested with the top roller approaching the sample with a displacement rate of 3.41 and 10 mm/min. The raw data associated with this graph is present in the data repository associated with this article. Flexural modulus was calculated in excel with $E_{B}=\frac{L^{3}m}{4bd^{3}}$ where *L* = support span (meters), *m* = slope of displacement-load curve, *b* is the width of the sample (meters), and *d* is the thickness of the sample (meters) [7]. The 10 mm/min samples had a flexural modulus of 2.86 GPa ± 0.151, and the 3.4 mm/min samples had a flexural modulus of 2.94 GPa ± 0.032 which are acceptable values for the current skull model. The MATLAB script *‘Skull_10mm’* was used to analyze the file for Fig. 6a, *‘100%Grid_PLA_3PB-3.4mm-min’* and Fig. 6b, *‘100%Grid_PLA_3PB_10mm-min’* [6]. Both of these files are available in the data repository titled *‘3-Point Bend Testing for 100% Grid Infill PLA Samples’* [6]. Although the MATLAB script specifies 10 mm, it is a general script that can assess samples of any speed setting.

**Fig. 6a:**
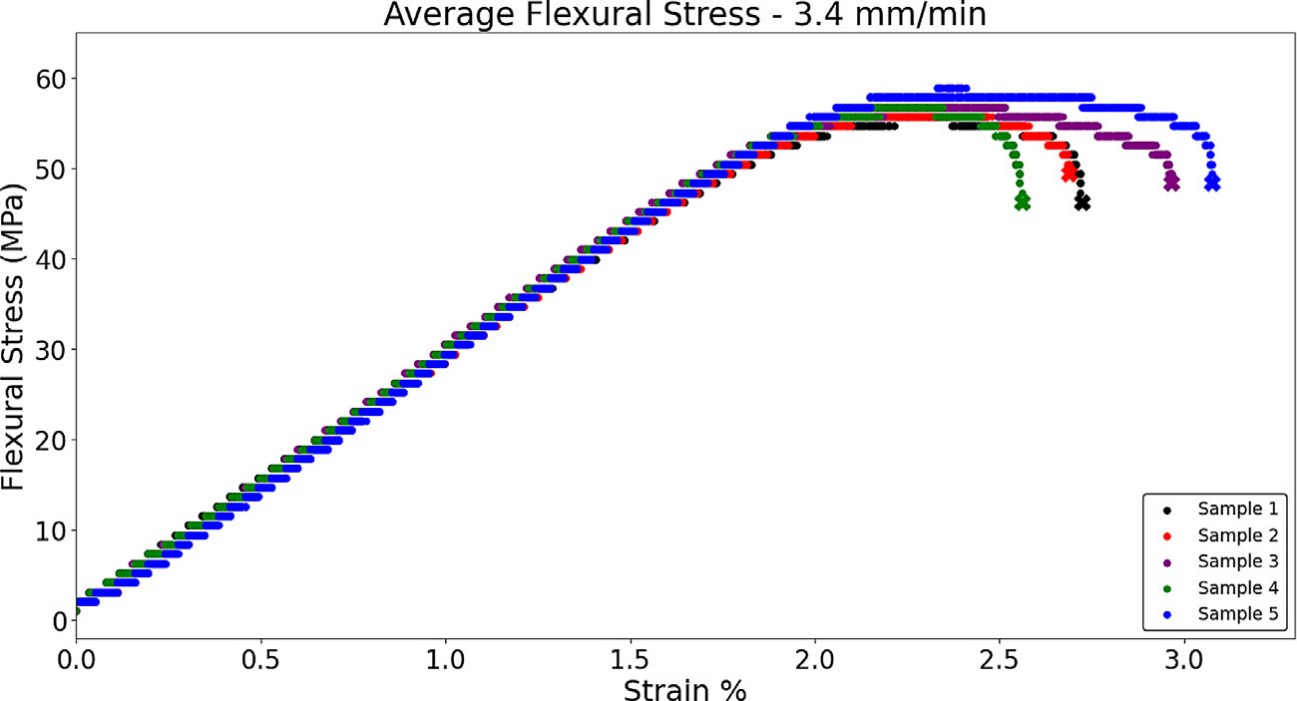
Flexural stress of PLA samples in 3-point bend testing performed at 3.41 mm/min

**Fig. 6b:**
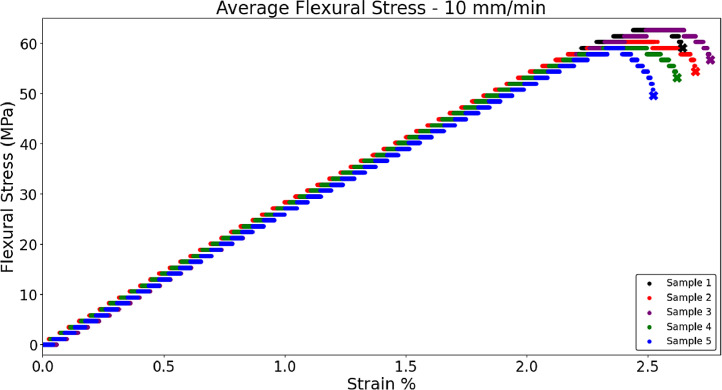
Flexural stress of PLA samples in 3-point bend testing performed at 10 mm/min

## 3 Experimental Design, Materials and Methods

### 3.1 Hydrogel Formulations

Four hydrogel variables were tested, monomer to crosslinker ratios (60-1 and 15-1), 5 – 12 wt. % monomer content, hydrogels with linear acrylamide chain content, and hydrogels polymerized at 60°C. The formulations with varied monomer content and monomer to crosslinker ratio followed the same basic protocol, deionized (DI) water is degassed and stirred for 10 minutes. Then acrylamide, methylenebisacrylamide (MBA), and the initiator ammonium persulfate (APS) are all added independently and degassed with a vacuum pump, and stirred for 3, 10, and 2 minutes respectively. Tetramethylethylenediamine (TEMED), acting as a catalyst, is added to the gel precursor solution to induce polymerization at room temperature. As an example, for a 100 mL batch of 10 wt. % 15-1 hydrogel 10 grams of acrylamide, 0.667 grams of MBA, and 0.0867 grams of APS are used with 180 μL of TEMED [10]. Monomer content is based on weight per volume and MBA is calculated based on desired crosslinker ratio, which in this example is 15-1. The APS and TEMED values are calculated with the following equations based on values used by Wermer, A., et al. (2020) [10].

$$APS\;\left(grams\right)=\left(8.67*10^{-5}\right)*Acrylamide\;\left(grams\right)*DI\;water\;\left(mL\right)$$

TEMED(μL)=0.178*Acrylamide(grams)*DIwater(mL)

### 3.2 Heated Hydrogel Formulations

Heated hydrogels were made with a similar protocol, DI water was heated, degassed, and stirred for 10 minutes. Acrylamide and MBA are added simultaneously and heated, degassed, and stirred for three minutes. After this period degassing and stirring is stopped and the temperature is brought to 60°C, then APS and TEMED are added simultaneously and stirred for 10 seconds. During all of this, the designated molds are heated to 60°C in a furnace. The gel-precursor solution is then added to the mold and placed back inside the furnace during polymerization.

### 3.3 Linear Acrylamide Hydrogel Formulations

Linear acrylamide hydrogels were prepared in two phases, the first is to prepare a linear acrylamide solution and the second is to polymerize the hydrogel. DI water is degassed and gently stirred for 10 minutes, then acrylamide is added based on the desired wt. % and degassed and gently stirred for 3 minutes. The solution is then heated to 37°C then APS and TEMED are added to form linear chains. The linear chain solution is maintained at 37°C and stirred for 2 hours. After that time, the solution is cooled to room temperature and stored in 4°C until needed. For the second step, hydrogel polymerization, DI water is degassed and gently stirred for 10 minutes, and then acrylamide, MBA, linear acrylamide chains, and APS are added and degassed independently while being stirred for 3, 5, 3, and 2 minutes respectively. TEMED is added and the solution is allowed to gel for 24 hours.The acrylamide, MBA, APS, and TEMED values are based on the same values indicated in 2.1. The same amount of APS and TEMED are added for both the linear solution and the hydrogel polymerization. The equations for these values were indicated in ‘2.1 Hydrogel Formulations’.

### 3.4 Rheological Measurements

The SR5 was utilized to conduct oscillatory rheology on hydrogel samples. Swollen hydrogel discs with a 25 mm diameter and approximately 1 mm height were polymerized and swollen in an isotonic solution for 24 hours. The upper fixture of the instrument was lowered onto the sample to obtain an axial force between 0.01 – 0.2 N. The stress setting for frequency sweeps was set under the yield stress of the sample, which was estimated by performing a stress sweep and finding the stress value where 1% strain was achieved. Hydrogels with a monomer to crosslinker ratio of 15-1 were tested without any sandpaper present to prevent slippage, whereas 60-1 hydrogels were tested with sandpaper. This is due to the different surface characteristics present on the 15-1 and 60-1 hydrogels. Data is reported to 10 Hz as inertial forces dominate the data beyond that.

### 3.5 Python Script

The rheological data was plotted with a simple Python script. Raw data is entered into excel and the Python script references the excel file to plot the data. This script calculates and plots the average and standard deviation of all samples in a batch.

### 3.6 Hydrogel Swelling

Hydrogel formulations were polymerized in cubic silicon molds approximately 3 cm in all dimensions. Hydrogel cubes were then removed from their molds and placed into an isotonic solution to swell. Cubes were measured with digital calipers and the average volume was calculated.

### 3.7 3D Printed Samples

PLA samples were 3D printed with a Creality CR-10S Pro V2. The settings of the printer were a 205°C nozzle temperature and 40 mm/s print speed. The samples were made to have a geometry of 154 mm length, 12.7 mm width, and 8 mm thickness. In this geometry the wall count, top, and bottom layer count were all set at 2. These samples had an infill percentage of 100% with a grid pattern.

### 3.8 3-Point Bend Testing

The specimens were manufactured and tested via three-point bending according to the ASTM D790-10 standard which tests flexural properties of polymers [7]. The three-point bending tests were conducted using a Mark-10 ESM1500S at a crosshead speed of 3.4 mm/min and 10 mm/min. Each specimen was tested until break or ultimate flexural strength (UFS) was reached. The flexural modulus is obtained from the average of the five specimens. In excel the flexural modulus was calculated by calculating the slope of the linear portion of the flexural stress.

### 3.9 MATLAB Script

Methods and calculations were determined by ASTM standards [7]. The raw data from the 3-point bend testing apparatus is obtained and entered into excel. Load versus displacement curves is plotted from the output data from the mechanical tester.

In the MATLAB flexural stress of the samples are calculated by:

$$\sigma_{f}=\frac{3PL}{2bd^{2}}$$

where *σ_f_* is the flexural stress, *P* is the load at a given point (N), *L* is the support span (mm), *b* is the width of the sample (mm), and *d* is the thickness of the sample (mm).

Flexural strain is calculated in the MATLAB script with:

$$\varepsilon_{f}=\;\frac{6Dd}{L^{2}}$$

where *ε_f_* is the strain, *D* is the maximum deflection (mm) at the center of the sample, *d* is the thickness of the sample (mm), and *L* is the support span (mm).

### 3.10 Optical Clarity Assessment

Hydrogels were polymerized in cuvettes with a sample thickness of 1 cm and tested in an Evolution 260 Bio UV-vis spectrophotometer from 400-700 nm at 2 nm intervals [11]. Slit width was 5 mm and integration time was set at 0.3 seconds. Light absorbance readings were obtained and averaged to compare light absorbance.

## Ethics Statements

The authors have read and confirmed the work in this article meets the ethical requirements necessary for publication in Data in Brief. This study does not involve any data collected from human subjects, animal experiments, or social media platforms.

## CRediT authorship contribution statement

**Anthony J.A. Baker:** Conceptualization, Methodology, Software, Investigation, Resources, Data curation, Writing – original draft, Writing – review & editing, Visualization, Supervision. **Eric J. Galindo:** Conceptualization, Methodology, Investigation, Resources, Data curation, Writing – review & editing, Visualization, Supervision. **James D. Angelos:** Methodology, Investigation, Resources, Data curation. **Dustin K. Salazar:** Software, Formal analysis, Data curation, Writing – original draft. **Sorcha M. Sterritt:** Validation. **Adam M. Willis:** Conceptualization, Writing – review & editing, Funding acquisition. **Michaelann S. Tartis:** Conceptualization, Methodology, Investigation, Resources, Writing – review & editing, Supervision, Project administration, Funding acquisition.

## Declaration of Competing Interest

The authors declare that they have no known competing financial interests or personal relationships that could have appeared to influence the work reported in this paper.

## Data Availability

Hydrogel Rheology, Optical Values, and Swelling (Original data) (Mendeley Data) 3-Point Bend Testing for 100% Grid Infill PLA Samples (Original data) (Mendeley Data)
